# How to Get Up Early

**Published:** 1878-06

**Authors:** 


How to Get Up Early.—Place a basin of cold water by the side of your bed; when you first awake in the morning, dip your hands in the basin and wet your brow, and sleep will not again seal you in its treacherous embrace.

This is the advice given by an aged man, who had been in the habit of rising early during a long life. By attending to this advice, you may learn to rise every morning at *five o'clock*. The Editor has found it to be a better plan to go to bed at one regular hour. Leave your bed the moment you awake of yourself, after daylight; nature will thus regulate the sleep to the exact amount required by the system.

